# Obsessive-compulsive spectrum – review of the construct

**DOI:** 10.1192/j.eurpsy.2023.1968

**Published:** 2023-07-19

**Authors:** S. Torres, J. Moura, J. F. Cunha, A. Lopes

**Affiliations:** Departamento de Psiquiatria e Saúde Mental, Centro Hospitalar Barreiro-Montijo, Lisbon, Portugal

## Abstract

**Introduction:**

Obsessive-compulsive disorder (OCD) is a clinical syndrome whose hallmarks are excessive, anxiety-evoking thoughts and compulsive behaviours that are generally recognized as unreasonable, but which cause significant distress and impairment. OCD may also occur in the context of other neuropsychiatric disorders, most commonly other anxiety and mood disorders. The question remains as to whether these combinations of disorders should be regarded as independent, cooccurring disorders or as different manifestations of an incompletely understood constellation of OCD spectrum disorders with a common aetiology.

**Objectives:**

To review critically whether there is a robust basis for the concept of an obsessive–compulsive (OC) spectrum of disorders, and if so, which disorders should be included.

**Methods:**

Literature review performed on PubMed and Google Scholar databases, using the keywords “obsessive–compulsive disorder”, “obsessive–compulsive spectrum”, “body dysmorphic disorder”, “hypochondriasis”, “trichotillomania”, “psychiatry”.

**Results:**

Obsessive–compulsive disorder (OCD) itself is a heterogeneous condition or group of conditions, and this needs to be appreciated in any articulation of a ‘spectrum’ of OC disorders. The basis for ‘membership’ of the spectrum is inconsistent and varied, with varying level of support for inclusion in the putative spectrum.

**Conclusions:**

A more fruitful approach may be to consider behaviours and dimensions in OCD and OC spectrum disorders, and that this should be encompassed in further developments of the OC spectrum model.

**Disclosure of Interest:**

None Declared

